# Engaging Black and Latinx Communities in Building Capacity for Behavioral Health

**DOI:** 10.3390/bs16081337

**Published:** 2026-08-04

**Authors:** Hoa B. Appel, Mabel C. Ezeonwu, Damari Peralez-Long, Leonor Bejarano, Dan Hudson, Roy L. Szeto

**Affiliations:** 1 School of Medicine, University of Washington, Seattle, WA 98195, USA; 2 School of Nursing and Health Studies, University of Washington, Bothell, WA 98011, USA; 3 Latino Educational Training Institute, Everett, WA 98204, USAbejarano.leonor@gmail.com (L.B.); 4 Seattle African American Men’s Civic Group, Seattle, WA 98118, USA

**Keywords:** capacity building, Black, Latinx, behavioral health, mental health, underserved, COVID-19, healthcare access, engagement

## Abstract

Introduction: COVID-19 affected nearly every aspect of people’s lives worldwide. In the United States, the consequences of closures and quarantine increased stress, worry, isolation, and mental health and substance use problems, leading to behavioral health (BH) problems. These conditions were exacerbated among Black and Latinx populations by language barriers, lack of mental health access, shortage of mental health providers, inadequate or lack of insurance coverage, and the health care system’s unfamiliarity with Black and Latinx cultures and consequent Black and Latinx distrust in it. Methods: The purpose of this project was to engage with Black and Latinx populations; connect with existing networks of community stakeholders, health providers, and researchers to co-learn about patient-centered outcomes research; and build partnerships with those that work with the communities. The engagement process involved regular focus group meetings and interviews with key stakeholders. Results: The key study’s outcomes resulted from an alliance of patients, caregivers, family members, community, and other stakeholders who learned about behavioral health issues of concern while prioritizing a research agenda. Discussion: The community-based participatory research approach from our study helped guide and build capacity for future patient-centered research among Black and Latinx communities.

## 1. Introduction

Prior to the COVID-19 pandemic, marginalized communities experienced disparities in health and other outcomes, many of which were exacerbated by the global event (Brooks et al., 2022). As COVID-19 quickly grew into a pandemic, every age group in the U. S. was impacted by school closures, home quarantine, isolation from friends, families, and coworkers, business closures, changes in employment (lack of work and work from home), and consequent impacts on personal relationships (Czeisler et al., 2020; Tull et al., 2020; Ye, 2020). These changes cascaded into an increase in stress, worry, isolation, and mental health and substance use problems (Alonzi et al., 2020; Zhou et al., 2020). For the reasons above, these challenges were heightened within Black and Latinx (BAL) populations, and this was our starting point.

Communities of color are exposed to greater risk of adverse mental health effects during the pandemic (Falicov et al., 2020; Zhou et al., 2020). Together with substance use problems, depression and anxiety took their toll during the pandemic (Alonzi et al., 2020; Usher et al., 2020), and other health-related disruptions were expected to continue long after the pandemic ended (Spence et al., 2023). Those already experiencing mental health challenges suffered intensified stresses (Alonzi et al., 2020; Czeisler et al., 2020). While the COVID-19 pandemic has ended, its consequences persist among underserved and vulnerable populations, particularly Blacks and Latinx (Ryu et al., 2025).

The Centers for Disease Control and Prevention (Centers for Disease Control and Prevention, 2021) report that, compared to White individuals, Black or African American individuals were 1.1 times more likely to be diagnosed with COVID-19, but 2.8 times more likely to require hospitalization and 1.9 times more likely to die (Centers for Disease Control and Prevention, 2021). Latinx (Hispanics and Latino/a) individuals are twice as likely to be diagnosed, three times more likely to be hospitalized, and 2.3 times more likely to die from COVID-19 compared to Whites (Centers for Disease Control and Prevention, 2021). Resources to support mental health and to address mental illness are often difficult to access due to a shortage of mental health providers, long waits for appointments, and inadequate insurance coverage (Health Resources Services Administration, 2025; Zao et al., 2018) In addition, when resources are available, Black and Latinx tend to underuse them due to unfamiliarity with the health care system, likely related to the fact that many providers do not match the demographic makeup of the communities they serve (Coulter et al., 2020; Pugh et al., 2021).

Finances and financial insecurity are additional stressors. Many lost jobs or reduced work hours, which significantly impacted their personal and family life, resulting in increased substance abuse and domestic violence (Donnelly & Farina, 2021; Ganson et al., 2021). Many Blacks and Latinx community members found that the increase in isolation led to mental health problems such as anxiety, depression, and suicidal ideation (Boserup et al., 2020; Hertz & Barrios, 2021; Horigan et al., 2021). Washington State presents an example of these effects.

In Washington State, the two most populated counties are King and Snohomish counties. This study focused on the BAL communities in these two Washington counties, including the stakeholders representing and serving these populations. The 2020 population estimates show that in King County, 7% of the population is Black or African American, and 10% is Latinx (United States Census Bureau, 2021). In Snohomish County, 4% are Black or African American and 10.6% Hispanic or Latinx. Overall, Washington’s population is estimated to be 13% Latinx and 4.4% Black or African American (United States Census Bureau, 2021). COVID-19 rates in Washington State disproportionately burdened these communities, with confirmed cases among Latinx individuals 3.5 times more frequent than among White individuals and twice as frequent among Black populations as among White populations (Washington State Department of Health, 2022). Metropolitan Seattle (in King County) comprises 30% of the State’s population and has the highest population density in Washington State (United States Census Bureau, 2021). The data underline the increased severity of the pandemic’s impacts, especially on underrepresented populations.

Research also demonstrates the importance of recognizing that mental distress experienced during the pandemic may lead to long-term mental illness, which can hinder personal, professional, and social relationships and activities (Appel & Nguyen, 2020; Mah et al., 2016; Steinert et al., 2015). Also of importance is the disproportionate impact on these communities on their economic and social well-being (Islam et al., 2021; Tipirneni et al., 2022). Families are affected by unemployment, and children face difficulties not being able to attend school, and food insecurities are some effects of the pandemic, especially among populations of color (Brown et al., 2020; Couch et al., 2020; Wolfson & Leung, 2020). This underscores the need to develop and implement strategies for capacity building to support behavioral health wellness among underserved communities.

Although COVID-19 wreaked various forms of havoc on all people, the common experience also provides an opportunity to collaborate and to learn how to best build capacity for the underrepresented communities of BAL populations. The overall goal of the study was to hear, understand, and acknowledge the voices of BAL members concerning their experiences before and during COVID-19, the response to the pandemic, and the behavioral health problems that arose and were exacerbated by the pandemic. The study directly engaged and entered into partnerships with BAL populations, and with existing networks of BAL community stakeholders, providers, and researchers to learn cooperatively about patient-centered outcomes. Research shows self-reported anxiety, depression, stress, and other negative mental health outcomes of COVID-19, especially for those who were forced to stay at home (Tull et al., 2020; Usher et al., 2020; Zhou et al., 2020). However, because the pandemic was new in 2020, the effects of some of our responses to it were not anticipated. Some requirements such as social distancing, which may have helped in decreasing the spread of the disease, also carried unintended deleterious mental health consequences (Banerjee & Rai, 2020; Miller, 2020). Our study examines the lived experiences of those psychologically affected during and after the pandemic so that research can be prioritized to address each community’s needs.

## 2. Materials and Methods

### 2.1. Objectives

The primary objective of this project was to build adequate capacity to support and provide ongoing support for the behavioral health needs of underserved BAL communities in King and Snohomish counties, the two most populous counties in Washington State. Our project’s specific aims and goals: (1) Establish two patient, family, and caregiver groups, one from each participating county. We convened two groups of BAL members, including patients and such stakeholders as families, caregivers, leaders of community organizations, and providers. These groups investigated priorities and issues of concern from the perspectives of these stakeholders. The goal was to hear each community’s and stakeholders’ concerns and experiences, especially regarding the BH challenges that arose during COVID-19, to identify the barriers and challenges they encountered, and the lingering mental health effects of the pandemic with eight convenings. (2) The second objective was to create an opportunity for BAL and stakeholders to co-learn about the research process from various perspectives, and (3) the last objective was to identify issues affecting BAL and the relevance of evidence-based practices through open and exploratory conversations with BAL and stakeholders to build capacity for future research. Necessary to this end is a deeper understanding of how the pandemic has impacted the mental health of those who are part of racial/ethnic minority groups. The goal of capacity building is to equip patients, caregivers, clinicians, and other stakeholders with the foundational knowledge and competencies to build infrastructure for future research.

### 2.2. Engagement Proposal

Research is well served by engaged participants. The quality of engagement results from group activities and in turn shapes the outcomes for the community and the project (Forsythe et al., 2018; Maurer et al., 2022). Researchers, participants, and community partners collectively engage in planning, facilitating and carrying out the project throughout the research life cycle. Engagement was grounded using the nominal group technique (Delbecq & Van de Ven, 1971). This technique uses small groups which help to facilitate open discussion and reach consensus. Small groups allowed inclusiveness of all members to engage in discussions and ensured that all voices were heard without any individual overpowering the conversation.

The group’s engagement included short-term outcomes during the project period. Among them were the formation of an alliance of patients, caregivers, family members, community and other stakeholders to learn about behavioral health issues of concern and prioritizing a patient-centered research agenda based on what the project team learned. This was based on PCORI’s (Patient Centered Research Institute) engagement rubric in engaging patients and community partners in research (Curtis et al., 2012). The rubric includes the six principles of engagement as part of the model: reciprocal relationships, co-learning, partnership, trust, transparency, and honesty (Curtis et al., 2012; Kauffman et al., 2013). The rubric provided a foundation to guide our engagement and was helpful in assessing the impact of engagement and our study.

Other engagement activities included evaluating how to best learn from each other about issues of importance to the communities with whom the project team collaborated. These activities provided a forum for participants to share their experiences during the pandemic, co-learn about the research process, and share their experiences at the end of the project by presenting what they learned from the project. To best examine the factors that influenced the mental health of community members during and after COVID, we used a mixed-methods study. We used discussions with the participants in small and large groups, surveys to learn about the engagement and content discussed, and semi-structured interviews with the clinicians, which allowed for rich data from different data sources. Our outcomes will help to build capacity for future research with patients, clinicians, and community stakeholders.

### 2.3. Procedures

We examined participants’ perceptions of what they encountered during and after COVID by asking all participants about their experiences. We prompted our participants to discuss barriers they faced, especially from economic and mental health perspectives. The mixed-methods study included interviews with health providers as well as data gathered from in-person meetings and discussions, which provided us with rich data from participants’ perspectives (Creswell & Clark, 2017). Our framework was based on community-based participatory research (CBPR), a valuable means of engaging community stakeholders in planning, recruiting, and implementation. This improves the feasibility, acceptability, and interpretation of research findings (Oetzel et al., 2018; Ortiz et al., 2020). CBPR engenders more comfort for participants with familiar community settings, encouraging them to feel more involved in the study, actively participate, and build upon strengths and resources of other participants and community partners (Kandula et al., 2008; Koya & Egede, 2007; Wallerstein & Duran, 2010).

Ethical approval was obtained from the University of Washington Institutional Review Board (STUDY 00015958). Participants either signed a paper consent form or an online consent form via DocuSign. Participants were informed that the study’s purpose was to understand the impact of the pandemic on participants and their families. Next, we engaged with our research team members, a process that included meetings with community leaders to learn how to recruit participants and whom to include on our research team. The final study team included the principal investigator (PI), co-PI, research assistant, and two community co-leaders as part of the research team. The research team met to discuss further recruitment methods, a timeline, and how to best engage participants. Participants were recruited through word of mouth, referrals from the project’s community co-leaders, healthcare providers, and community leaders between September and October 2022. Participants’ informed consent was obtained by the start of the first meeting in November 2022.

Regular meetings to engage BAL community members, providers, researchers, and other stakeholders were held to identify issues of concern. We conducted interviews with health care leaders and clinicians to co-learn research principles and prioritize research agendas to build capacity. In months when we did not meet with study participants, we checked in by phone and Zoom with these participants (Table 1). These practices enabled us to address mental health concerns that arose during COVID-19, and also those that arose thereafter.

We developed participant groups, each led by a group leader. Meetings occurred on Zoom and in person monthly. The core team of group leaders, the PI, co-PI, research assistant, and two community co-leaders met monthly on Zoom to plan the study’s goals and coordinate every other month meetings with study participants. All team members and study participants completed the PCORI’s Fundamentals of Research online to ensure all participants understood the basics of research (PCORI, n.d.). We also interviewed six clinicians from the two counties via Zoom. The clinicians included a psychiatric nurse practitioner, a hospital administrator, and four clinicians who provided behavioral health care.

Prior to our first meeting, all participants completed an online pre-survey. The purpose of the pre-survey was to gather demographic information, behavioral health issues encountered during and after COVID, and participants’ expectations of the study. The post-survey was administered online at the last meeting. In-person participants completed the survey at the end of the meeting, while the Zoom participants completed it before signing off from the meeting. The post-survey gained information about participants’ perceptions of the meetings, the online check-ins when we did not meet in person, and the overall content learned in the study.

In order to keep track of our Black and Latinx participants and ensure our stakeholders were up to date on study activities, we developed a study website with a blog. The website contained group information and project activities, such as progress notes, bimonthly meeting information, finalized research agenda, and information on the final group report. Our project website was created with input from participants and team members and was maintained by the website designer. The website allowed participants to see the viewpoints and activities from both groups. Our project participants were encouraged to share information about their participation in the study on social media as a way to demonstrate their valuable input, hard work, and value to the team. We offered the option to use our blog should our BAL participants and stakeholders also prefer to use it. The study’s website designer assisted with the website, blog, and all online activities, including orientation of participants under this project.

There were seven in-person convenings for all participants, which occurred bimonthly. In addition, we had seven individual telephone and/or Zoom check-ins to hear from participants, for a total of 14 meetings. In the last meeting, everyone met to discuss the study’s results and the next steps for the sixteen months of the study. The remote check-ins permitted us to learn from participants how the study was proceeding, if there were any questions or concerns about the study, or suggestions for it. We took the comments and suggestions into consideration at each bimonthly check-in with participants.

### 2.4. Participants

Participants were recruited based on community co-leaders’ connections in their respective Latinx and Black community organizations. Recruitment was based on word of mouth, flyers emailed to various communities where participants frequent, and online referrals via emails. To be included in our study, participants had to be at least 18 years old, able to speak English, and willing to share their mental health experience during and after COVID. Also, eligible participants had to be minority patients, caregivers, or clinicians residing in either of the two counties where we recruited them. The sample size and composition were determined by feasibility and availability, contributing to a more diverse sample with a broadened population and without any selection bias.

Recruitment prioritized Black and Latinx participants because of the study’s interest in understanding their experiences. However, participation was also extended to individuals from other racial and ethnic groups who met the study’s substantive eligibility criteria and had direct experience with the phenomenon under investigation. These participants were retained as information-rich cases within a heterogeneous qualitative sample. The analysis examined experiences across the sample while remaining attentive to racial and ethnic context; it was not designed or sufficiently powered to make subgroup comparisons. The relevant literature supports heterogeneous sampling only when the participants share the central phenomenon and their inclusion serves the research aim. Purposeful and maximum-variation sampling as approaches for selecting information-rich cases has been supported by the literature (Palinkas et al., 2015), while Malterud et al. (2016) explain that qualitative sample adequacy depends on “information power,” not sample size alone.

All participants were interviewed and screened for eligibility, including willingness to share their experiences during and after COVID-19, participation in group discussions online and in-person, and sharing at the end of the study’s community conference. Informed consent was requested when participants agreed to participate in the study and prior to the start of the first meeting. The meetings occurred from November 2022 to June 2024. Participants were compensated with a $100 gift card for each of the in-person meetings. In addition, we interviewed six clinicians from King and Snohomish counties to learn their perspectives in providing care to patients during and after COVID.

**Figure 1:**
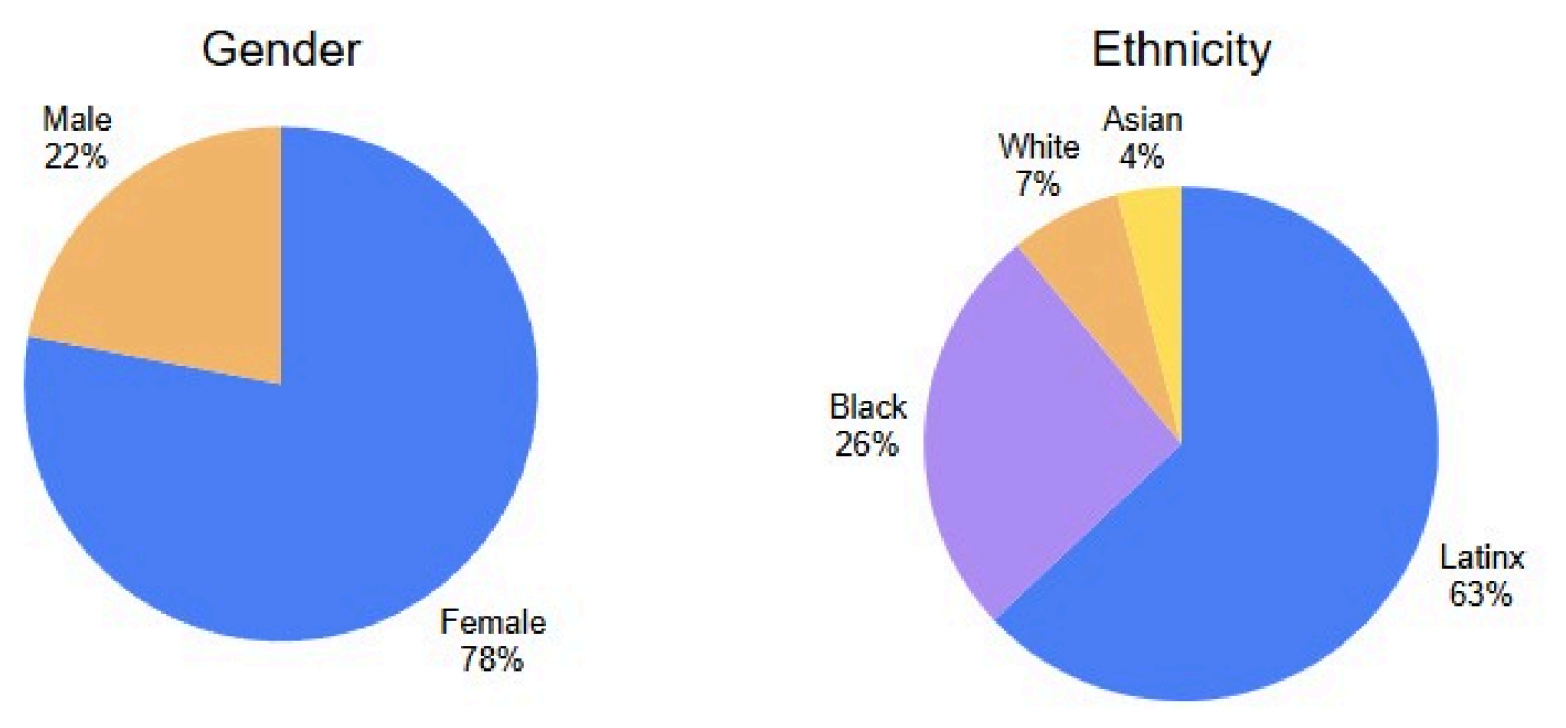
Participant demographics (*N = 27*).

Figure 1 shows the study’s composition of 27 participants, with the majority being Latinx (63%) and Black (26%). The remainder were White (7%) and Asian (4%). Even though we had a majority of Blacks and Latinx participants in the study, the remaining 11% were also heard and provided a good comparison with the other two groups. There were 21 females and 6 males. All participants were patients during and after COVID; 18 were caregivers in their respective families, and two were community stakeholders. We also interviewed six clinicians who saw patients during the pandemic.

### 2.5. Instrument

Each participant completed a pre-survey on engagement, working collaboratively in groups, and behavioral health issues encountered during the pandemic. This included qualitative questions and some Likert-scale questions on how the pandemic was affecting them. The mid-study survey contained qualitative questions based on how group interaction progressed to that point, how best to work together for the remainder of the study, and how COVID-19 affected their personal and professional lives, including mental health. The post-survey contained the same questions as the pre-survey, with the added question on participants’ takeaways from the study’s discussions and whether their perspectives on mental health had changed because of participating in the study.

### 2.6. Data Analysis

#### Qualitative Data

The in-person meetings and virtual check-ins yielded rich qualitative data. Themes and interpretations were collected, analyzed, and discussed collectively among the research team with input from all participants in the following in-person meetings. To identify themes from the qualitative data, we used thematic analysis to identify, describe, and organize themes from the group discussions and interviews, which give voice to participant experiences (Braun & Clarke, 2006; Nowell et al., 2017). The analysis was conducted by a graduate student who began by reading the transcripts to learn about concepts and discuss repeat themes. Then, a codebook was developed based on discussions. Codes were created to identify new themes arising from participants. The research team reviewed the initial codebook and modified it after discussion and feedback. The refined codebook was coded independently by the graduate student, with groupings of codes organized into themes and subthemes, along with selected quotes that aligned with the themes and subthemes. The coder used Excel to complete the organization of data, retrieval and stratification.

## 3. Results

### 3.1. Quantitative Findings

At the end of the study, we re-administered the survey that we had administered mid-study, as described above, to compare what participants thought about the study at the mid-survey with what they thought at the end. The post-survey revealed that all participants found the study to be helpful. The post summary showed an increase in all categories from the initial engagement process (Table 2). The survey is based on a Collaboration Assessment Tool (CAT) that we modified to apply to our group (Marek et al., 2015). The original CAT questions were modified for use in this study. We did not ask questions about coalitions that participants belonged to because this was not applicable. We also excluded duplicate and similar questions from the seven key factors shown in Table 2. Due to the small sample size, we included all participants in our analyses.

The CAT allowed measurable qualitative and quantitative data on effective collaboration. Our highest mean scores were from “Members bring unique skills in the way the group works”, “The community provided me with support and opportunity to share my personal experiences and the struggles of communities of color”, and “Team leaders support and help us to work together as a team”. 

Table 2 also shows the development of group engagement and collaboration from the time of the mid-study survey to post-completion of the study. The highest mean scores were from “The community provided me with support and opportunity to share my personal experiences and the struggles of communities of color”. Also, the majority of those surveyed indicated “the group provides a safe environment in which members can share personal information, especially about mental health”.

We conducted a non-parametric analysis to evaluate the changes between Time 1 and Time 2. Because the data is ordinal and involves a small sample size, we used the Wilcoxon signed-rank test for paired participants to determine statistical significance. Table 2 shows statistically significant improvement from T1 to T2 in a variety of measures, including “all members participate in decision-making” and “Team leaders support and help us to work together as a team”.

### 3.2. Qualitative Findings

In the in-person meetings, we discussed examples and ideas of how we would reach consensus to understand the participant groups’ interaction and communication, to identify questions that could be answered, and to identify research modules to learn about the appropriate research process and principles of research. Additionally, we discussed each individual participant’s goals as well as the group’s goals; some of those were learning about culturally sensitive issues and crafting appropriate research questions. Each member of the research group had relevant, individual lived experience. Accordingly, our co-learning yielded meaningful shared group goals. Our process of co-learning ensured we kept in mind the guidance we received from the participants and stakeholders. Our collaboration presented opportunities for every member of our project to participate and gain from that experience.

We focused on reviewing and discussing existing research literature while integrating the lived experiences of the participating members and community leaders who work with BAL populations to create the research agenda. This required prioritization by the group. The resulting process was based on the nominal group technique, which provides a structured mechanism for objectively prioritizing key issues adopted by the group (American Society for Quality, n.d.). We then ranked the group’s ideas, which allowed members to reach consensus in participant-centered research. The resultant research agenda established a foundation to better understand the experience and needs of the BAL. Among other things, the agenda included how to engage and pursue opportunities to conduct future research, and to develop new ways to share information learned.

In addition to continued conversations and discussions with patients and stakeholders, we interviewed six clinicians and one hospital administrator. These interviews were fruitful, yielding information consistent with the comments of patients in our group. For example, clinicians indicated their concern over the lack of minority healthcare providers serving minority communities, the change from in-person to virtual health, and healthcare workers retiring during the pandemic due to burnout. The clinicians mentioned that COVID-19 presented a good opportunity for conversations about mental health. They noted the need for change to increase access to healthcare for all, especially the underserved, financially disadvantaged, or people of color.

Our study participants have access to all study materials available online so that they can continue to share ideas among themselves and act cooperatively as appropriate. Participants worked together and in groups of up to three people and presented their group’s selected project at the last community conference. Our website designer continued to support those less familiar with the online platforms. Participants had access to all the study’s materials, could collaborate/track ideas through a Google Doc, or link to a survey. 

As a result of the group’s collaborative effort, we developed a plan to disseminate the project’s results to our community partners and work with them to carry out its recommendations. We plan future research in this area. Our study team hopes to sustain our collaborative effort. In particular, we hope to develop a grant proposal to address the behavioral health issues brought up during our project, especially those affecting BAL communities. Our final formal meeting, a community conference to share the results of this study, was held in person and on Zoom, at a local library. It was an opportunity to awaken the community to issues affecting the BAL communities.

**Figure 2:**
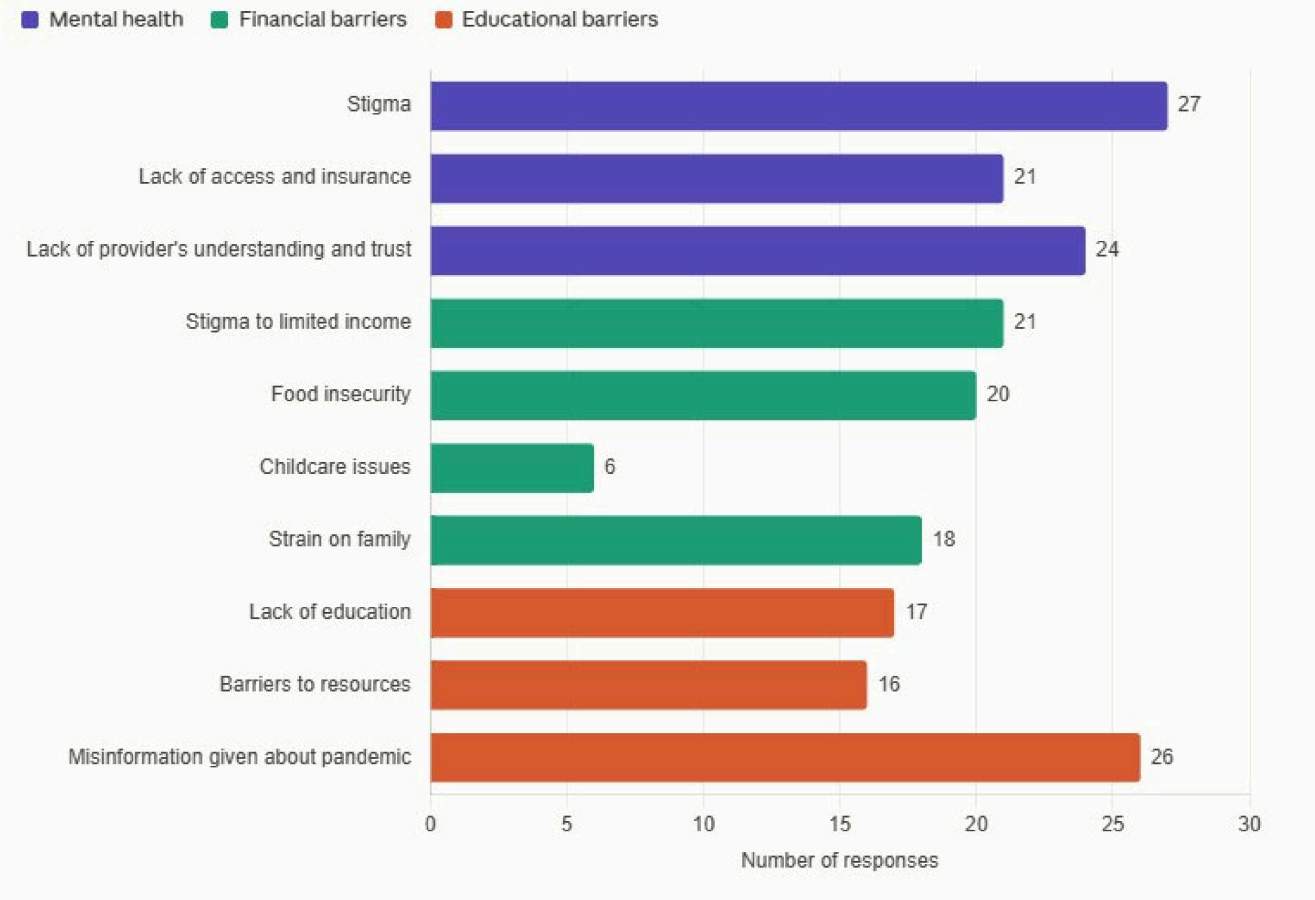
Themes and sub-themes identified through group discussions and number of participants who mentioned sub-themes.

The thematic analysis showed individual responses from group discussions and bi-monthly online check-ins. Community building and a sense of belonging from participants were key to the sharing of lived experiences. Three key themes emerged from the participants’ input (Figure 2).

First, mental health was a major element of the participants’ concerns. This included barriers to receiving mental health care and the stigma of mental/behavioral health care. With mental health care are included the subthemes of insurance issues, healthcare access, and provider trust issues.

Second, participants raised the matter of financial barriers due to lack of employment or work hours. Financial barriers led to participants having to prioritize necessities such as food, rent, utilities, or insurance, to the exclusion of mental health care needs. Financial barriers also led to food insecurity, impacting physical and mental health.

Lastly, educational barriers were impacted during the pandemic. Misinformation regarding healthcare and mental health impacted the participants’ daily lives and interaction with their families. Also, educational barriers impacted how participants received care, with cultural barriers and responsiveness not being addressed.

One positive outcome that ameliorated mental health and cultural barriers was the group’s cohesiveness due to its members’ similarity of lived experiences.

*“Everyone went through different things or different challenges—food insecurity, mental health. The community aspect was very rewarding to connect with different people. Focus groups like this to focus on ways on how to invite communities to work together for future resources and services regarding mental health or access to resources regarding mental, social, and physical health.”*

(Participant 3)

*“Hearing all of these different experiences is like a mirror and learning from others in more ways we can help. Learning how all of those experiences connect to the experiences.”*

(Participant 7)

*“I enjoyed the collaboration, especially in person, being in the presence of others instead of online, especially after the pandemic. It’s nice to be with others and sharing ideas; pushes me out of my comfort zone and to improve my speaking skills.”*

(Participant 10)

Healthcare providers that were interviewed shared their points of view from caring for patients during the pandemic. Systemic barriers in the healthcare system as well as lessons learned were prominent during and after the pandemic:

*“Our healthcare system has significant systemic challenges that were exacerbated or exposed more due to the pandemic. People of color face significant socioeconomic barriers that have direct effects on their ability to seek care or manage a chronic condition. Our healthcare workforce has been battered by the pandemic, and still faces some structural challenges related to recruitment and retention moving forward.”*

(Provider 1, Nurse Practitioner)

*“My suggestions on supporting the mental health needs of vulnerable populations are that we need more mental health professionals, and mental health training for family members.”*

(Provider 3, Family Physician)

### 3.3. Strengths of the Engagement

This study used the nominal group technique to engage group members (Delbecq & Van de Ven, 1971). Groups were divided into smaller groups of 5–7 participants. The technique is a structured decision-making and brainstorming method designed to generate ideas. Proposed solutions are prioritized, and then the group reaches consensus after everyone has had an opportunity to participate. After small group discussions, we regrouped, and everyone returned to the large group to share what they learned in the small group discussions. The technique was useful in including all group members in the engagement process, thereby permitting equity in the decision-making process. This consideration reinforces the importance of inclusion and equity factors for this project. All voices were heard, and everyone had an equal chance to be engaged in group decisions with their own contributions to the engagement process. The results showed the positive impact that group engagement had from the feedback shown in Table 2.

## 4. Discussion

We examined perceived barriers from populations of color during and post-COVID. The mental health and social impacts from the pandemic disproportionately affected families from low-income and racial and ethnic minority groups. As noted in previous research, our participants shouldered a disproportionate burden of both the prevalence and social challenges related to the pandemic, including those related to children, food insecurity, and unemployment (Brown et al., 2020; Couch et al., 2020; Falicov et al., 2020; Wolfson & Leung, 2020).

As a result of the in-person and Zoom meetings, participants could engage in a comfortable space to share their behavioral health challenges during COVID. As mentioned above, we were pleasantly surprised at the openness of sharing, especially by those with mental illness and caregivers of family members. Also, some participants mentioned they enjoyed participating in research, learning more about their selected topic, and adding excellent, from-the-heart information on mental health to their personal stories. 

Our original plan was to have two study groups, one from each county. However, participants and stakeholders agreed to meet as one group at a central location near the border between the two counties. This worked out well because it led to one large, cohesive group and fewer separate meetings. Also, we were able to hear from participants with similar and different concerns depending on where they lived. We met our other study’s aims, including creating an opportunity for Black and Latinx members to co-learn about patient-centered research from various perspectives. Another objective that we completed was the identification of relevant, evidence-based issues affecting these communities.

Even though we planned during the grant application process as well as during preparation for our first convening, our engagement activities and planning around those activities adapted and improved as the project progressed. We added an amplifier and external speaker to supplement our laptop so that the Zoom group could hear better. Not all remote participants had their cameras on during our hybrid meeting and, as we expected, we saw less engagement from the remote group than from the in-person group. The small breakout rooms helped, but we always had better engagement from the in-person group. From the interpersonal engagement that occurred throughout the project, we were able to co-learn about the impact of COVID-19 that affected the participants, their families, caregivers, clinicians, and the community at large. One comment from a caregiver summed up one of the accomplishments nicely: “This project has opened my mind and perspective on ways COVID has brought light to mental health and racial disparities.” The open discussion of mental health issues encountered is important to show participants who experienced similar disruptions and consequent mental health problems due to the pandemic are in good company. This builds solidarity, especially among racial and ethnic minorities (Brown et al., 2020; Islam et al., 2021; Wolfson & Leung, 2020; Zhou et al., 2020).

Throughout much of the project, we were on time with our deliverables, participants were helpful with the planning for the community conference (CC), and in what we consider an organic success, post-study items revealed some participants wanted to do more research with the study team. Participants were energized and encouraged to continue to be involved. Some remain in contact with the study’s research team to further the work developed from the project. Out of all this, the research team put together a presentation guideline that many said helped them focus on their topic and presentation technique. Additionally, the study team formulated a CC Summary, which was distributed during the CC to all participants and community members whom study participants had invited to that conference. The CC Summary contains the key points discussed during the study and some possible next steps for a future research application.

The post-survey revealed all participants found the study to be helpful. The post summary showed an increase in all categories from the engagement process and confirmed that the study team provided support and opportunity for participants to share their lived experiences and struggles during and after the pandemic. The survey allowed measurable qualitative and quantitative data on effective collaboration. The highest mean scores were from members who felt that they brought unique skills in working in a group. Our findings confirm that patient-centered communication is key to informing best practices and policies and helps to improve trust in healthcare services and providers (Cuevas et al., 2019; Lucock et al., 2015). Also, we learned that the CBPR approach contributes to deeper engagement, allows all participants to be equally engaged, and benefits the community through research. It also builds a sense of empowerment among community members as well as creating opportunities for action to improve health (Oetzel et al., 2018; Ortiz et al., 2020; Wallerstein & Duran, 2010).

The study did not merely help on an individual level. The information gained was also valuable in the participants’ respective communities. For example, one patient noted that, “the mental health issues really affect all of us and the power we have as a community when we work together to achieve a common goal”. Another participant shared, “There is a lot of strength in sharing experiences. I find the more people talk about mental health, it encourages other people to talk about mental health.” One participant aptly summed up the study, saying, “This really was a project that brought us together from different worlds and recreated one world together. I really appreciate how down-to-earth and real things were… We shared a lot of resources that a lot of us didn’t know were out there.”

Overall, the study allowed for participants and community members to co-learn about each other’s experiences during and post-COVID. Participants said they felt comfortable sharing their experiences; that they learned more about themselves and healthcare resources. Lastly, the study served as capacity building for future patient-centered research with a larger sample size that would include patients, caregivers, clinicians, and other stakeholders to engage and improve behavioral health care for underserved populations.

### Limitations and Future Research

Our study has a few limitations. First, the sample size was small, and we lacked a control group. Also, more females than males participated in the study. This is a weakness, as female experiences were different from those of males. However, our work was designed mainly as a pilot study focused on community-based participatory research and hearing about the experiences of the community members affected by COVID.

Second, although the sample predominantly included Black and Latinx participants, it also included a small number of Asian and White participants. Their inclusion provided additional perspectives on the shared phenomenon but limits the extent to which the pooled findings can be interpreted as specific to Black and Latinx populations. The small subgroup sizes also precluded meaningful comparisons by race or ethnicity.

Third, we encountered some challenges due to lost consultants. We managed to overcome them with our research team members and by keeping our participants and stakeholders appraised of the changes as well as obtaining their input on how to meet the challenges. One of our Latinx consultants moved his professional practice and so became unable to be our consultant. Fortunately, our Latinx community liaison volunteered to add more hours to the project and consulted with her supervisor to help with recruitment. She also came up with engaging activities at our hybrid meetings.

Fourth, recruitment and retention of participants were a challenge. Some participants dropped out for various reasons. Some moved, one was unable to continue for the remainder of the study, another went back to school, and yet another said the mental health topic affected participation. We were unable to replace those who dropped. Our goal was to have a waitlist in anticipation of the attrition, but no one signed up to be on the waitlist. This was because the duration of the project seemed too long for some. We tried to recruit more during the first two months of the project’s start but were only able to add one more participant by referral from a member of the study group. In addition to the improvement needed in participant retention, future studies should also consider expanding the sample size by expanding the geographical area of recruitment and adding a one-year post-study follow-up to assess the effectiveness of that community engagement among the participants and community organizations.

Despite our small sample size, our quantitative and qualitative findings provide a valuable and unique contribution to the limited research on both Black and Latinx populations from their own experiences during the pandemic. Our engagement process guided us to participant-centered outcomes and practices that will be used to submit a future research project using outcomes relevant to these communities. Overall, the convenings with robust discussion and engagement demonstrated promising results for future research on how these populations felt shaped by the constraints exacerbated by COVID-19. These findings should be explored further with a larger sample size of underserved populations.

## Figures and Tables

**Table 1:** Description of in-person meetings, check-ins, and timeframe.

In-Person Meeting Activities	Remote Meeting Activities	Duration	Session Date
Introductions, discuss study’s goals, expectations, and timeline		2 h	November 2022
	1st remote check-in: to answer questions and hear feedback	20 min	January 2023
Co-learn about research methods and practice research skills		2 h	March 2023
	2nd remote check-in: update and Q & A from participants	20 min	May 2023
Semi-structured interviews and discussions to evaluate collaborative effort using the Collaborative Assessment Tool (CAT)		2 h	June 2023
	3rd remote check-in: update and research modules Q & A	20 min	August 2023
Discuss and develop issues affecting participants during and post pandemic		2 h	September 2023
	4th remote check-in: contribute to project’s blog and community conference (CC) introduction	20 min	October 2023
Create, discuss, and prioritize future research agenda from issues of concern		2 h	November 2023
	5th remote check-in: follow up from last month’s meeting on research agenda	20 min	January 2024
Create strategic plan to build capacity for future research		2 h	February 2024
	6th remote check-in: follow up from last month’s meeting; CC prep	20 min	March 2024
Plan details for Community Conference including venue, whom to invite, materials to share with community.		2 h	April 2024
	7th remote check-in: Q & A about community conference	20 min	May 2024
Final preparation for CC, discussed project’s summary and sharing of group’s lived experiences		1 h	June 2024
Community Conference to promote awareness of community members, stakeholders and researchers of research agenda created participants.		2 h	June 2024

**Table 2:** Group functioning ratings at mid-program and post-program (*N = 24*).

Description	Mid Mean (*SD*)	Post Mean (*SD*)	*z*	*p*	*r*
**Topics related to group members**					
Members share an understanding and respect each other.	4.72 (0.53)	4.88 (0.33)	−1.26	0.206	−0.48
Members are willing to compromise (recognize decisions cannot fit the preferences of every member perfectly).	4.32 (0.88)	4.63 (0.63)	−1.54	0.124	−0.49
Members understand the roles, rights, and responsibilities of all members.	4.36 (0.84)	4.54 (1.04)	−0.91	0.363	−0.27
Members bring unique skills to address this group’s needs.	4.68 (0.61)	4.96 (0.20)	−1.93	0.053	−0.73
Members feel ownership in the way the group works.	4.12 (0.95)	4.25 (0.83)	−0.74	0.457	−0.20
All members participate in decision-making.	4.20 (0.75)	4.58 (0.64)	−1.97	0.049 *	−0.51
**How the group works**					
Strategies to carry out the goals of the group are clearly explained.	4.56 (0.75)	4.54 (0.64)	−0.50	0.617	−0.14
Members select or are assigned roles and responsibilities according to their interests and strengths.	4.36 (0.84)	4.54 (0.71)	−0.37	0.712	−0.10
A system of communication is in place for members to discuss their efforts.	4.36 (0.79)	4.71 (0.61)	−1.54	0.124	−0.49
**Group communication**					
I get enough communication from group leaders about what’s happening.	4.48 (0.64)	4.58 (0.64)	−0.90	0.366	−0.27
This group provides a safe environment in which members can share personal information, especially about mental health.	4.64 (0.74)	4.88 (0.44)	−1.39	0.165	−0.52
Communication among members is effective (promotes understanding, cooperation, and sharing of information).	4.52 (0.64)	4.67 (0.75)	−0.71	0.477	−0.21
**Group functioning**					
This study group has clearly defined goals and objectives.	4.44 (0.70)	4.63 (0.70)	−1.51	0.132	−0.45
Members agree upon the goals and objectives for this study group.	4.56 (0.64)	4.67 (0.47)	−0.63	0.527	−0.20
The goals and objectives set for this study group can be realistically achieved.	4.48 (0.81)	4.71 (0.54)	−1.16	0.244	−0.32
Members view themselves as working together to achieve the goals and objectives of this study group.	4.52 (0.70)	4.71 (0.61)	−0.88	0.377	−0.27
**Group leadership**					
Research team leaders are prepared and well organized for each meeting.	4.48 (0.76)	4.67 (0.55)	−1.07	0.284	−0.31
Team leaders are able to work well with each other in addition to working well with different personalities in the group.	4.64 (0.63)	4.83 (0.37)	−1.89	0.059	−0.71
Team leaders support and help us to work together as a team.	4.68 (0.61)	4.92 (0.40)	−2.12	0.034 *	−0.95
Team leaders carry out the role with fairness, making sure all voices are being heard and respected.	4.68 (0.68)	4.88 (0.33)	−1.15	0.248	−0.38
Team leaders maintain a focus on the goals and objectives of the study group.	4.64 (0.63)	4.92 (0.28)	−2.11	0.035 *	−0.75
Team leaders offer support to members outside of meetings.	4.12 (1.03)	4.75 (0.60)	−2.35	0.019 *	−0.65
**Collaborative success (1 = not confident, 5 = very confident)**					
How successful is this study group at achieving its goals?	4.40 (0.69)	4.63 (0.63)	−1.50	0.134	−0.42
How confident are you that this study group will continue to successfully achieve its goals?	4.52 (0.64)	4.63 (0.63)	−0.81	0.417	−0.27
How confident are you that this study group will continue to make a difference within the community it serves?	4.64 (0.63)	4.75 (0.52)	−0.58	0.564	−0.19

Note. Items were rated on a 5-point scale (1 = strongly disagree, 5 = strongly agree), except items in the Collaborative success domain (1 = not confident, 5 = very confident). z and *p* are from Wilcoxon signed-rank tests; *r* = rank-biserial correlation (effect size). Negative *z* and *r* values reflect the direction of the signed-rank computation; all changes were toward higher post-program ratings. * *p* < 0.05.

## Data Availability

The data are available upon request to the corresponding author.
